# Elective splenectomy for hematological diseases: a vanishing indication

**DOI:** 10.1007/s00464-024-11071-8

**Published:** 2024-08-29

**Authors:** Sonia Fernández-Ananin, Silvana Novelli, Lorena Cambeiro Cabré, Cecilia Vila Riera, Eulalia Ballester Vàzquez, Elisabet Julià Verdaguer, Eduardo M. Targarona

**Affiliations:** 1 Universitat Autònoma de Barcelona (UAB), Medical School, Upper Gastrointestinal and Bariatric Surgery Unit, Department of General and Digestive Surgery, Hospital de la Santa Creu i Sant Pau, Sant Quintí 89, 08041 Barcelona, Spain; 2 Surgery Research, Institut de Recerca Sant Pau (IR Sant Pau), Barcelona, Spain; 3 Hematology Department, Medical School, Hospital de la Santa Creu i Sant Pau, Universitat Autònoma de Barcelona (UAB), Barcelona, Spain

**Keywords:** Laparoscopic splenectomy, Hematological diseases, Autoimmune diseases, Medical therapies, Immune thrombocytopenic purpura

## Abstract

**Introduction:**

Splenectomy has been used as a diagnostic and therapeutic tool in the management of hematological diseases for many years. However, the emergence of new medical therapies has modified guidelines for many hematological diseases for which splenectomy was previously considered. We aimed to evaluate the evidence of a decrease in the hematological indications for splenectomy and the reasons and justifications for this change.

**Material and methods:**

We conducted a single-center, retrospective analysis of patients who underwent laparoscopic splenectomy for hematological disease between January 2010 and December 2023. Patients were classified into four groups: 1 autoimmune and hemolytic diseases (HAD), (2) lymphomas, (3) myeloproliferative diseases (MPN), and (4) other splenic diseases. We recorded the annual incidence of splenectomy and the ratio of new medical cases, demographic and clinical data and surgical outcomes.

**Results:**

During the study period, 98 patients were referred for splenectomy. There was a significant progressive decrease in this surgical indication, particularly regarding HAD (*p* < 0.001). The indication for splenectomy for immune thrombocytopenic purpura (ITP) declined to zero despite an increase in the number of patients diagnosed with this disorder (*p* < 0.001). The pattern of decrease in AHAI and Evans syndrome was similar to that in ITP. The group of splenectomies due to lymphoma persisted consistently during the study period, as did the indication for splenectomy in the context of lymphoma treatment. Splenectomy due to massive splenomegaly secondary to MPN was indicated only in one patient. Splenectomies due to other causes were similarly distributed over the years.

**Conclusions:**

Our findings confirm a significant decrease in the indication for elective surgery for hematological diseases, mainly regarding autoimmune disease. The surgical community and surgical departments should be aware of this situation yet maintain the skills to adopt this technique both safely and efficiently.

Splenectomy has long been a useful diagnostic and therapeutic tool in the management of several hematological diseases [1, 2] and may be used as a therapeutic option in refractory cases to medical treatment. A diagnostic splenectomy may be required when there is a high suspicion of malignancy or when staging the disease. A splenectomy may also be indicated for palliative purposes in cases of induced hypersplenism or clinical discomfort secondary to massive splenomegaly.

The main drawback of conventional open splenectomy is its intrinsic complications, particularly the lifetime risk of overwhelming sepsis, portal vein thrombosis, or pulmonary hypertension [3, 4]. The introduction of the laparoscopic approach significantly reduced the surgical injury, thereby decreasing postoperative pain and the risk of infection, and enabling a faster recovery. Laparoscopy also relaunched interest in this surgical option, particularly for frail patients and patients with massive splenomegaly [5]. However, simultaneously, over the 30 years since the introduction of laparoscopic splenectomy (LS), we have seen the introduction of novel medical therapies, new diagnostic imaging methods, biological markers, and better diagnostic protocols. Such changes have led to modifications in the guidelines and treatment recommendations of many hematological diseases for which splenectomy would have been an option [6–10].

Over these last 30 years, with an institutional series of over 500 cases, we have had a special interest in the evolution of the minimally invasive surgical approach to the spleen in our surgical unit [5], but in recent years we have observed a constant decrease in the annual number of new cases of hematological diseases referred for splenectomy. The current incidence of elective splenectomy for the treatment of hematological disease in an average surgical service is low, with only 6–10 cases performed yearly. The fading of surgical indications due to the advance of new medical therapies has not been an infrequent phenomenon in recent decades for some surgical treatments, such as peptic ulcer, portal hypertension, achalasia, bile duct stones, and the “watch and wait” option for rectal cancer.

This study aimed to examine the evidence of a reduction in the hematological indication for splenectomy and to evaluate the reasons and justification for this decrease.

## Material and methods

We conducted a single-center retrospective study of a prospective database of cases of splenectomy performed for hematological diseases in the surgery department at a tertiary referral center. We included all patients referred for splenectomy due to, or with the suspicion of a hematological disease between January 2010 and December 2023. Medical and surgical records were systematically reviewed. All information was obtained according to local data protection laws and regulations and the study was approved by the hospital ethics committee.

The series of elective splenectomies were subclassified into four groups: (1) autoimmune and hemolytic diseases (HAD), which included immune thrombocytopenia (ITP), autoimmune hemolytic anemia (AIHA), Evans Syndrome (ES) and spherocytosis; (2) lymphomas; (3) myeloproliferative neoplasms (MPN), and (4) other splenic diseases (cyst, metastasis, splenic tumors). We did not include cases of splenectomy to treat malignant disease (local invasion of the gastric or colon tumors) or cases of pancreatic tumor or splenic trauma.

We recorded demographic, clinical, and analytical parameters, the indication for splenectomy, previous medical therapy, immediate surgical outcome, conversion, length of stay, morbidity, and mortality according to the Dindo-Clavien classification [11].

Simultaneously, we recorded the global number of acute HAD in need of treatment, newly diagnosed lymphomas, and MPN diagnosed during the same period to determine the population at risk or susceptible to splenectomy over time.

A descriptive statistics analysis was carried out, and the comparison between two proportions (Student *T* test and Chi-squared analysis) was calculated. (Excel Microsoft 18.0) (Table 1).

**Table 1:** Splenectomy indications distribution (2010–2023)

Pathology	*n*	%
Group1: Autoinmune and hemolytic diseases	58	58
ITP	45	45
AIHA	7	7
Evans syndrome	5	5
Spherocytosis	1	1
Group 2: Lymphoma	15	15
Non-hodgkin lymph	15	15
Hairy cell leukemia	1	1
Group 3: Myelofibrosis		
Myelofibrosis	1	1
Group 4: Others	20	20
Splenic cyst	5	5
Haemangioma	5	5
Metastases	3	3
Primary malignant splenic tumour	5	5
Splenic abscess	3	3
Normal spleen	2	2
Total	98	

## Results

Between January 2010 and December 2023, 98 patients were referred to our department for splenectomy. Hematological diagnosis, patients´ demographic data, and surgical outcomes are described in Tables 2 and 3.

**Table 2:** Laparoscopic splenectomy outcome

Variable	Frequency (%)	Range
Sex	Male 41 (42%) Female 57 (58%)	
Age	56.1 (mean)	(17–86)
Op Time	102.56 (mean)	(60–240)
Conversion	1 (1%)	
Stay (d)	7.87 (mean)	(2–70)
Weight (gr)	505 (mean)	(37–3495)
Diameter (cm)	15 (mean)	(7–33)
Morbidity	33 (33%)	
Clavien I	9 (9%)	
Clavien II	14 (14%)	
Clavien III	9 (9%)	
Clavien IV	1 (1%) Late sepsis complication	
Mortality	1 (1%)	
Portal thrombosis	0	
Late septic morbidity	3 (3%)

**Table 3:** Evolution in the recommendation of Splenectomy for treatment of ITP in international guidelines during the last years (modified of Godeau [6])

Country/organization	Yr	2º line treatment	Period disease-LS (weeks)
USA	1995	splenectomy	4–6
UK/BCSH	2003	splenectomy	Not specified
USA	2010	splenectomy, TPO, Rtx for failed LS	6 or contraindication LS
Int Consensus	2010	SL vs Med Ther (Rtx, TPO-RAs)	12
Germany/Austria	2018	TPO Ras SL emergent situations	12
USA update	2019	SL vs Med Ther (Rtx, TPO-RAs)	12 personalized
Int Cons Update	2019	SL vs Med Ther (Rtx, TPO-RAs)	12–24 personalized
China	2019	SL vs Med Ther (Rtx, TPO-RAs)	6
Australia	2019	SL vs Med Ther (Rtx, TPO-RAs)	12 personalized, avoid SL > 65 yr
Japan	2019	SL vs Med Ther (Rtx, TPO-RAs)	not specified
Spain	2021	Med Ther (Rtx, TPO-RAs, Fosfo)	12 SL x rescue after failure Med Treat
Belgium	2021	SL vs Med Ther (Rtx, TPO-RAs)	12
Korea	2022	TPO-RAs vs SL	12

*Rxt* Rituximab, *TPO-Ras* thrombopoietin receptor agonists, *SL* splenectomy, *Med Ther* medical therapy

**Fig. 1:**
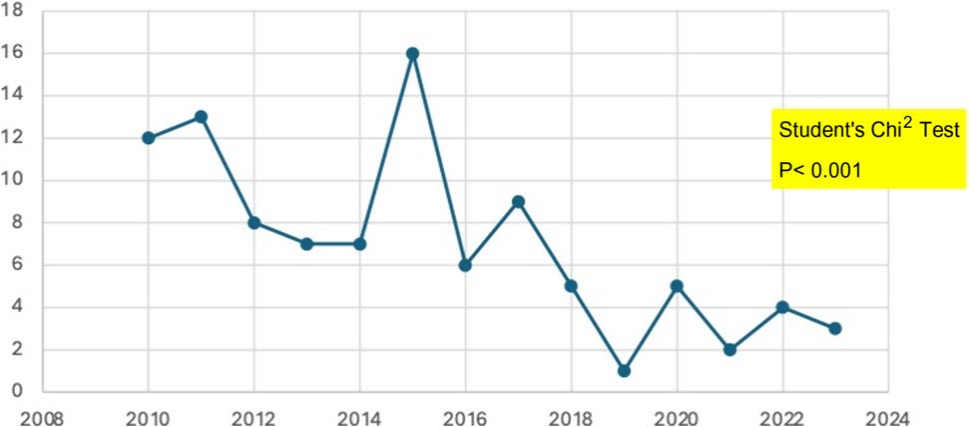
Overall declining incidence of yearly elective splenectomy for hematological diseases in relation to new diagnosed cases. (Chi^2^ *p* < 0.001)

During the 13 years of the study, we observed a statistically significant reduction in the overall number of patients that required splenectomy in relation to new hematological diagnosis in the overall series (*p* < 0.001), particularly due to the reduction in the indication of splenectomy in the Group 1 (AHD) (*p* < 0.001). We observed a progressive decrease in the indication for splenectomy for treating autoimmune diseases, even though immune thrombocytopenia remains the most frequent indication for this technique (Fig. 1).

**Fig. 2:**
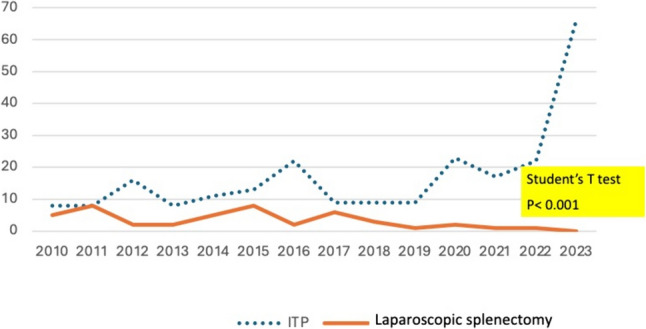
Declining incidence of yearly elective splenectomy for autoimmune hematological diseases (ITP) in relation to new diagnosed cases (Chi^2^ *p*: 0.001)

Group 1 included 58 patients, 45 of whom corresponded to ITP, 12 to other autoimmune diseases (5 ES and 7 AIHA) that were refractory to previous therapies, and a case of spherocytosis. Six cases were secondary to other hematological diseases. The indication for splenectomy in this subgroup of patients declined progressively to zero in the last years of the study despite an increase in the number of patients diagnosed with ITP during the same period (Fig. 2) (*p* < 0.01). In patients with ITP, LS was always performed after at least 2 lines of pharmacologic therapy (corticosteroids, thrombopoietin receptor agonists, or immunosuppressive agents).

The reduction in the indication of LS was also observed in AHAI and Evans syndrome, in a pattern similar to that noted in ITP cases (Fig. 2).

**Fig. 3:**
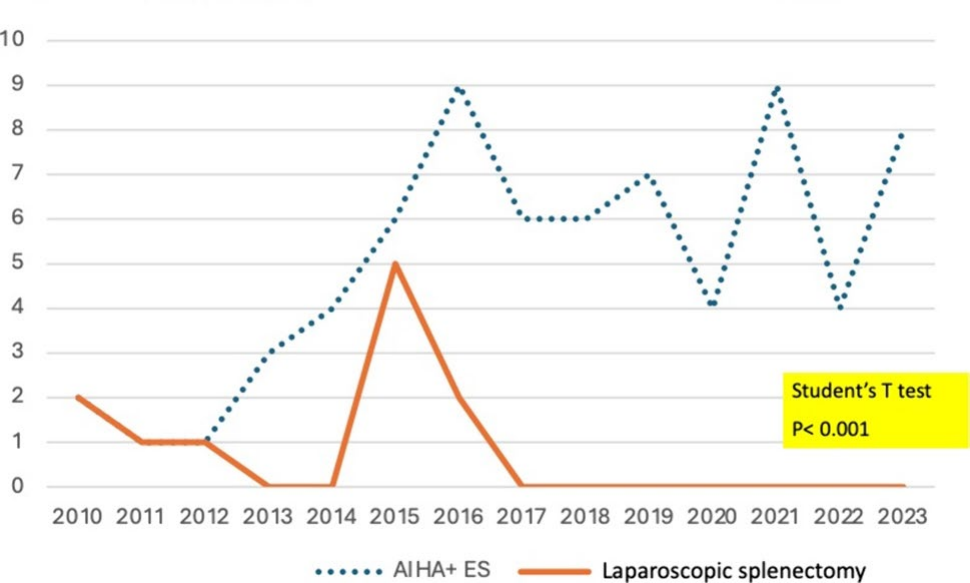
Declining incidence of yearly elective splenectomy for autoimmune hematological diseases (Evans syndrome and autoimmune hemolytic anemia) I relation to new diagnosed cases. (Chi^2^ *p* < 0.001)

The diagnosis of new cases of lymphoma (Group 2) persisted steadily during the study period, as did the low and scarce indication for splenectomy in the context of its treatment. In this subgroup of patients, splenectomy was performed in 16 patients. Splenectomy had a diagnostic-only purpose in six cases that resulted in high-grade lymphomas requiring additional chemo-immunotherapy. In the remaining cases (*n* = 12), LS was diagnostic of low-grade lymphoma, such as splenic marginal zone lymphoma, and the procedure was also therapeutic. Among the patients with marginal zone lymphoma (*n* = 10), 7 achieved remission, but 2 relapsed, at 1 year and 8 years, respectively (Fig. 3).

There was only 1 indication for splenectomy due to massive splenomegaly secondary to MPN (Group 3). In this case, the surgery was indicated to mitigate the abdominal discomfort and to reduce platelet transfusion.

Group 4 included 20 cases, with a similar distribution over the years (p = ns). There were 3 cases of metastases (two colon and one endometrial cancer), 3 splenic abscesses, 5 benign primary tumors, 2 malignant tumors (angiosarcoma) 2 normal spleens, and 5 cysts.

**Fig. 4:**
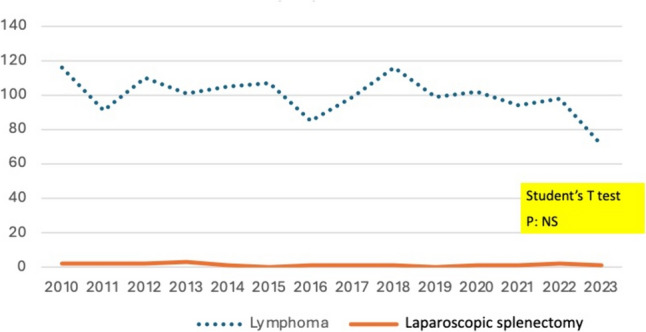
Maintained incidence of yearly elective splenectomy in relation to a new diagnosis of lymphoma (Chi^2^, *p*: NS)

In all 98 cases, the splenectomies were performed by the laparoscopic approach, and conversion to open surgery was needed in one case. Three cases of infection were observed (2 respiratory and 1 gastrointestinal sepsis). One death was related to the surgical procedure (Fig. 4).

## Discussion

The analysis of our experience over the last 13 years in a unit with a special interest in hematological surgery revealed a sharp decrease in the referral of patients for LS. We observed a progressive yearly reduction in referrals of over 70% from 2010 to 2023. This phenomenon has been widely identified, indicating the priority use of alternative medical therapies that avoid the need for therapeutic or palliative splenectomy [6, 12, 13]. Splenectomy continues to be of interest, however, for diagnosis, especially in selected cases of suspected lymphomas, and for therapy in cases of primary splenic tumors [14].

In our series, this reduction was especially evident in Group I, corresponding to autoimmune diseases (HAD). ITP has been the most frequent indication for LS for many years, and to date, international guidelines consider LS as a second-line therapeutic option after the failure of steroid therapy. However, the arrival of immunosuppressive such drugs as rituximab and thrombopoietin receptor agonists (TPO-RAs) (romiplostim and eltrombopag) has had an important impact [7, 8]. Despite the excellent response rates after splenectomy for ITP (> 85%), the emergence of new therapies with similar response rates (up to 80%) [7, 8] puts the indication for splenectomy under the scope. Moreover, TPO-RAs have reported long-term remission after treatment discontinuation in 30–50% of responders with a good safety profile, whereas splenectomy is an irreversible procedure that increases the risk of life-long infectious complications and portal vein thrombosis.

Evidence of a reduction in the surgical indication for ITP has been observed in clinical practice and the literature. Bhatt et al. [12] observed a decline over time [3.4% (2005–2006) to 1.6% (2013–2014), *p* < 0.001] in a series of 37.844 ITP hospitalizations from 2005 to 2014, with splenectomy performed in 954 encounters. Filianos et al. [13] evaluated the National (Nationwide) Inpatient Sample (NIS) in adult patients with a main diagnosis of ITP between 2007 and 2017. A total of 36.141 hospitalizations for ITP were included in the study. The splenectomy rate declined over time (16% in 2007 to 8% in 2017, trend p < 0.01). Simultaneously [6], in the prospective French CARMEN registry, which included 1.247 adult ITP patients in France, after a mean follow-up of 16.2 ± 18.7 months, only 17 (1.3%) of patients underwent splenectomy (Table 3). In Italy, Palandri et al. [14] studied the role of splenectomy in the therapeutic strategy of ITP between 1980 and 2015. They showed that 40 years ago, splenectomy was used as second-line therapy in 46% of cases, whereas between 2010 and 2015 it was merely 3%.

The indication for splenectomy has evolved from the second-line preferred option to an alternative when the current second-line or even third-line option fails, as observed in the statements and guidelines published in recent years [6–8]. This observation has also been observed in cases of autoimmune hemolytic anemia and Evans syndrome in a parallel way to that of ITP patients [9].

Our series includes only one case of splenectomy indicated for the treatment of spherocytosis. A reduction in the indication of splenectomy for this disorder has been also observed previously. In the experience of Vercellati et al. [10] from among 446 patients with hereditary spherocytosis diagnosed in the last 40 years in a reference center, the frequency of splenectomy decreased over time (from 44% before 1990 to 7% in 2011–2020) despite confirmed efficacy. Age at splenectomy progressively increased (63% in children before 1990 to 88% in patients aged ≥ 20 years in 2011–2020).

A splenectomy to establish the final diagnosis in cases of a suspected primary tumor or relapsing lymphoma is scarce, but it is in these cases that LS continues to be a useful and safe surgical option. During the study period, we observed a steady yearly incidence of new cases of lymphoma. The percentage of patients requiring diagnostic or therapeutic LS was also constantly low (below 2%). The role of splenectomy as a diagnostic approach has recently been revised [15]. It was assertive in 95% of cases, and the most frequent diagnosis was lymphoma 16/20 (70%). As we know, advances in flow cytometry and molecular biology of cancer allow the detection of tumoral cells and genetic material (liquid biopsy), thereby reducing the future necessity for tissue biopsy, especially in the relapse setting [16]. The need remains, however, for tissue analysis to establish a diagnosis according to the current classification to guide therapeutic intervention.

Another hematological disease in which a sharp reduction in the indication of splenectomy has been observed is myelofibrosis. In our experience and during this 13-year study period, only one patient, with a myeloproliferative disease, was referred for surgery. Splenectomy indications in the case of primary myelofibrosis were usually related to secondary hypersplenism and symptomatic splenomegaly. However, since the introduction of ruxolitinib, an oral Janus kinase (JAK) 1/JAK2 inhibitor that effectively reduces splenomegaly in 42% of patients and improves symptoms, the indication for splenectomy is highly improbable [17, 18].

Anecdotal indications for LS are secondary metastasis located on the spleen [19], with breast cancer, colon cancer, or melanoma being the most frequent diagnosis. This situation is infrequent usually because spleen metastasis in most cases is associated with disseminated disease, but in selected cases, LS could be a safe option. Another infrequent indication could be suspected primary splenic tumors in which the procedure would simultaneously be diagnostic and therapeutic. A primary splenic cyst is also a condition that benefits from therapeutic LS.

Analysis of the surgical outcome of the laparoscopic approach in our series of cases again demonstrates the benefits of this approach, including the very low conversion rate, short hospital stays, low morbidity, and the fact that it remains a feasible indication despite potentially adverse circumstances (low platelet count, splenomegaly).

The main limitation of this study is that it is a retrospective, single-center experience for a relatively infrequent surgical procedure. However, the risk of bias is limited because it is referred to absolute figures observed during the study period. However, we consider the results to be of interest for the surgical community because the sharp reduction in the indication for this procedure may convert it into an anecdotal or rarely performed surgery. Consequently, although it is not a technically challenging procedure for the well-trained endoscopic surgeon, specific clinical details such as low platelet count or splenomegaly may increase its difficulty.

This study’s take-home messages would be addressed to two groups of practitioners, the hematologist and the surgeons. Hematologists should be well informed about the current efficacy and safety of splenectomy by the surgeons interested in splenic surgery, despite the clear advantages of noninvasive therapies, discussing the right position of this old, risky but extremely effective therapy. Adequate inter-service discussions and continued information to the clinician about the potential advantages of surgical approaches in selected cases should be maintained. On the other hand. we consider the results to be of interest to the surgical community in two directions: (1) Inside the surgical group should exist surgeons interested and well-trained in hematology to be able to discuss and negotiate, surgical indications with the hematologists, and (2) Due to the sharp reduction in the indication for this procedure, it may be converted into an anecdotal or rarely performed surgery. Consequently, although it is not a technically challenging procedure for the well-trained endoscopic surgeon, specific clinical details such as low platelet count, or splenomegaly may increase its difficulty. The surgical community and surgical services should be aware of this situation and the need to maintain team skills to perform this kind of surgery.
